# Left Medial Liver Lobe Torsion Associated With Hepatocellular Carcinoma in a Pembroke Welsh Corgi: A Case Report

**DOI:** 10.1002/vms3.71261

**Published:** 2026-10-03

**Authors:** Hitomi Shinoda, Kenji Mori, Hiroshi Mori, Kumiko Ishigaki, Kazushi Asano

**Affiliations:** ^1^ Ohmihachiman Animal Medical Center, Mori Animal Hospital Shiga Japan; ^2^ Laboratory of Veterinary Surgery, Department of Veterinary Medicine, College of Bioresource Sciences Nihon University Fujisawa Kanagawa Japan

**Keywords:** computed tomography, dog, hepatic lobectomy, hepatocellular carcinoma, liver lobe torsion, surgery

## Abstract

Liver lobe torsion (LLT) is a rare, potentially life‐threatening condition in dogs and is reported most often in medium‐ to large‐breed dogs. Neoplasia‐associated LLT is unusual, and detailed descriptions of LLT associated with hepatocellular carcinoma (HCC) are scarce. A 12‐year‐old spayed female Pembroke Welsh Corgi (9.5 kg) presented with acute collapse after several months of progressive lethargy and inappetence. Laboratory abnormalities included increased alanine aminotransferase and aspartate aminotransferase activities and C‐reactive protein concentration. Computed tomography (CT) identified a 7.2 × 6.7 × 6.5 cm mass arising from the left medial liver lobe, a whirl sign at the base of the mass and a non‐enhancing hypoattenuating region consistent with necrosis. Midline celiotomy and left medial liver lobectomy were performed without detorsion. Cytology of abdominal effusion was consistent with a culture‐negative inflammatory exudate. Histopathology confirmed HCC with extensive necrosis, and the examined surgical margin was free of neoplastic cells. The dog recovered uneventfully, was discharged on postoperative Day 4 and had no recurrence of clinical abnormalities at 1‐year follow‐up. This case highlights the apparent association between HCC and LLT and the unusual location of the torsion axis at the base of the mass rather than at the hepatic hilus, although causality cannot be established from a single case. CT visualisation of the whirl sign at this atypical site was instrumental in establishing the preoperative diagnosis and guiding surgical planning.

## Graphical abstract

In a 12‐year‐old Pembroke Welsh Corgi, hepatocellular carcinoma was associated with left medial liver lobe torsion, with the torsion axis located at the base of the mass rather than at the hepatic hilus. Computed tomography revealed a whirl sign at this atypical site, enabling preoperative diagnosis and surgical planning. Left medial liver lobectomy without detorsion resulted in uncomplicated recovery, with no clinical or imaging evidence of recurrence or metastasis at 1 year.

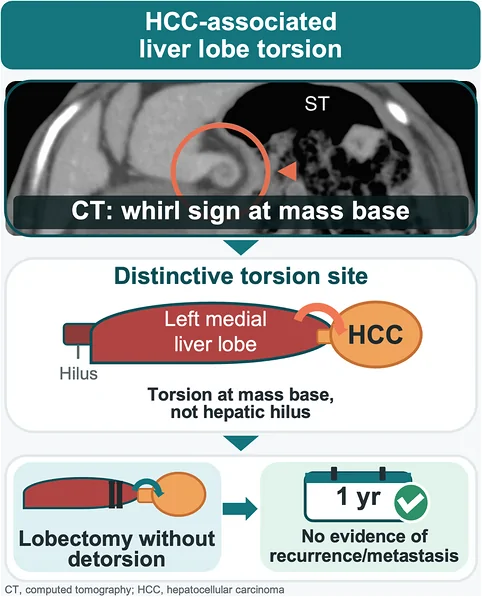

## 1 Introduction

Liver lobe torsion (LLT) is a rare condition characterised by rotation of one or more hepatic lobes around its vascular pedicle. The resulting venous obstruction can lead to hepatic congestion, thrombosis, ischemic necrosis, peritoneal effusion, shock, sepsis and death if definitive treatment is delayed (Swann and Brown 2001; Schwartz et al. 2006). In dogs, published information on LLT consists mainly of individual case reports and small retrospective series (Tomlinson and Black 1983; Downs et al. 1998; Swann and Brown 2001; Schwartz et al. 2006; Bhandal et al. 2008; Massari et al. 2012; Tubby 2013; Cordella and Bertolini 2021).

Ante‐mortem diagnosis is challenging because the clinical signs and clinicopathological findings are nonspecific. Reported signs include lethargy, anorexia, vomiting, abdominal pain, polyuria/polydipsia, peritoneal effusion and acute collapse (Schwartz et al. 2006; Khan et al. 2016). Medium‐ to large‐breed, middle‐aged to older dogs are overrepresented, and the left lateral liver lobe is most commonly affected, presumably because of its size, mobility and relative anatomical separation from adjacent lobes (Downs et al. 1998; Sonnenfield et al. 2001; Swann and Brown 2001; Schwartz et al. 2006; Bhandal et al. 2008; Lee et al. 2009; Tubby 2013). However, LLT has also been described in small‐breed dogs and puppies, indicating that signalment alone should not exclude LLT from the differential diagnosis (Sonnenfield et al. 2001; von Pfeil et al. 2006; Lee et al. 2009; Waskover et al. 2025).

Diagnostic imaging is central to preoperative diagnosis and surgical planning. Radiography and ultrasonography may identify a cranial abdominal mass effect, peritoneal effusion or absent blood flow in the affected lobe, but these findings are not specific. Computed tomography (CT) can identify the affected lobe, vascular compromise, non‐enhancing necrotic parenchyma and the whirl sign, which is highly supportive of torsion when present (Cordella and Bertolini 2021). Prompt liver lobectomy is considered the treatment of choice, and long‐term outcome is generally favorable in dogs that survive the perioperative period (Swann and Brown 2001; Schwartz et al. 2006; Scheck 2007).

Neoplasia‐associated LLT appears to be uncommon, and detailed reports of LLT associated with hepatocellular carcinoma (HCC) are limited. This report describes left medial LLT associated with HCC in a 12‐year‐old Pembroke Welsh Corgi and emphasizes the value of CT diagnosis, lobectomy without detorsion, histopathological confirmation and clinical follow‐up.

## 2 Case Presentation

### 2.1 History, Clinical Findings and Diagnostic Imaging

A 12‐year‐old spayed female Pembroke Welsh Corgi weighing 9.5 kg was presented for acute collapse after several months of gradually progressive lethargy and inappetence. The dog had no relevant previous medical history. On physical examination, the dog was dull and had mild abdominal pain. Rectal temperature was 38.0°C, heart rate was 140 beats/min and non‐invasive systolic blood pressure was 90 mmHg.

Complete blood count and serum chemistry revealed markedly increased alanine aminotransferase (ALT; 1228 U/L), aspartate aminotransferase (AST; 248 U/L) and C‐reactive protein (CRP; 6.0 mg/dL), as well as thrombocytosis (Plt; 678 × 10^3^/µL). Mild increases in total protein (TP) and globulin (Glob) concentrations were also present. Other measured variables were within the reference intervals (Table 1).

**TABLE 1:** Haematology and serum chemistry findings of the patient.

Parameter (unit)	Initial presentation	Postop 2 week	Postop 1 year	Reference interval
RBC (10^6^/µL)	8.57	8.04	7.90	5.65–8.87
PCV (%)	59	54	55	37–62
WBC (/µL)	12,920	14,900	7830	5050–16,760
Plt (10^3^/µL)	**678**	**508**	**498**	148–484
Glu (mg/dL)	117	109	109	70–143
BUN (mg/dL)	26	27	16	7–27
Cr (mg/dL)	1.1	1.0	0.8	0.5–1.8
ALT (U/L)	**1228**	**166**	76	10–125
AST (U/L)	**248**	**70**	47	0–50
ALP (U/L)	171	**215**	87	23–212
GGT (U/L)	5	5	3	0–11
TP (g/dL)	**8.3**	6.8	7.4	5.2–8.2
Alb (g/dL)	3.3	3.0	3.1	2.2–3.9
Glob (g/dL)	**5.0**	3.9	4.3	2.5–4.5
TCho (mg/dL)	158	156	196	110–320
TBil (mg/dL)	0.3	0.3	0.1	0.0–0.9
Na (mmol/L)	159	155	155	144–160
K (mmol/L)	4.5	5.1	3.8	3.5–5.8
Cl (mmol/L)	120	110	115	109–122
CRP (mg/dL)	**6.0**	**1.6**	0.4	0.0–1.0

*Note*: Bold values indicate results above the reference interval.

Abbreviations: Alb, albumin; ALP, alkaline phosphatase; ALT, alanine aminotransferase; AST, aspartate aminotransferase; BUN, blood urea nitrogen; Cr, creatinine; CRP, C‐reactive protein; GGT, gamma‐glutamyl transferase; Glu, glucose; Glob, globulin; NA; not analysed; PCV, packed cell volume; Plt, platelet count; RBC, red blood cell count; TBil, total bilirubin; TCho, total cholesterol; TP, total protein; WBC, white blood cell count.

Abdominal radiography revealed a large, well‐defined cranial abdominal mass with caudal displacement of adjacent viscera. Abdominal ultrasonography identified an approximately 7 cm mass between the left aspect of the liver and the spleen, with a small amount of anechoic peritoneal effusion. No detectable blood‐flow signal was observed within the mass on colour Doppler imaging. Doppler signals in the visible adjacent hepatic and splenic vasculature showed no obvious abnormality. The dog was hospitalised and medically stabilised.

The CT scan (Aquilion 16, Canon Medical Systems, Ohtawara, Japan) identified a 7.2 × 6.7 × 6.5 cm hepatic mass with a whirl sign at its base. On the complete multiplanar portal venous‐phase CT dataset, the twisted vascular pedicle was continuous with the portal venous branch supplying the left medial liver lobe. The lobar origin was subsequently confirmed intraoperatively. The mass contained a non‐enhancing hypoattenuating parenchymal region (15–22 HU), consistent with necrosis secondary to vascular compromise (Figure 1B,C). Mild abdominal effusion was present, and no pleural effusion was detected. These findings supported a diagnosis of left medial LLT (Figure 1A–C).

**FIGURE 1:**
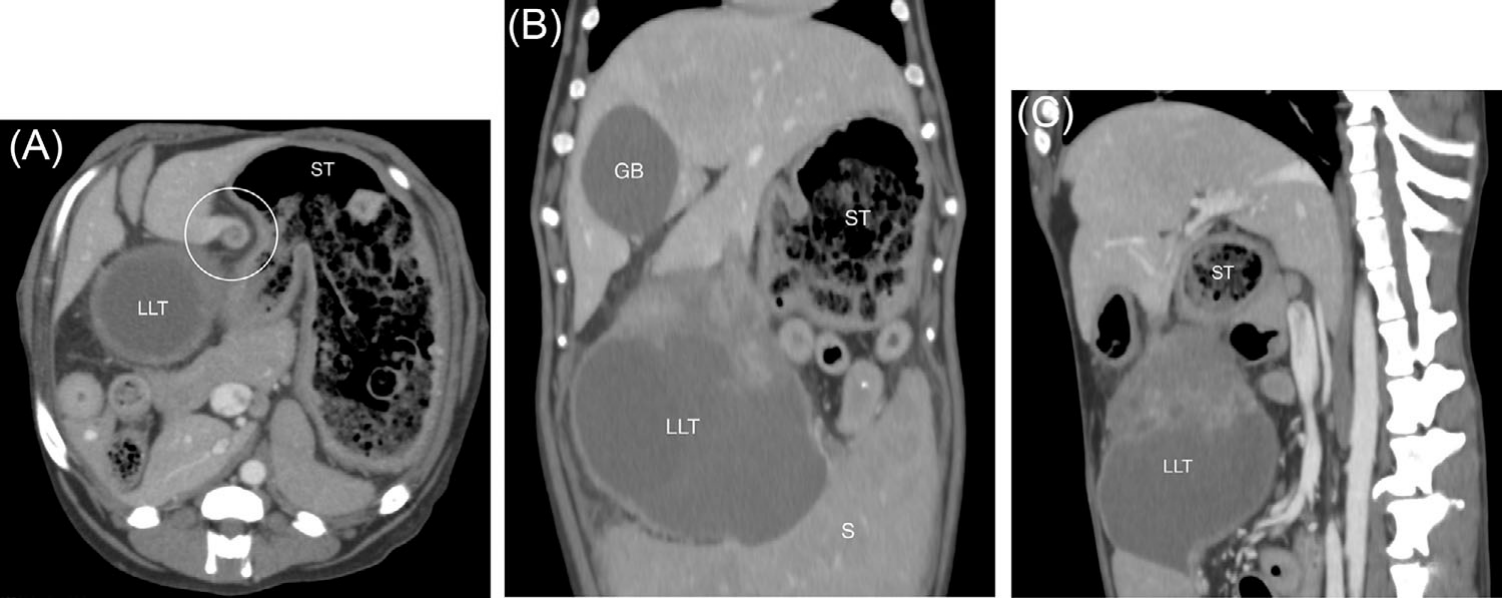
Preoperative computed tomography images (portal venous phase). (A) Transverse image showing a whirl sign at the base of the mass (circle), consistent with torsion of the left medial liver lobe. (B) Dorsal/coronal reconstructed image showing the hepatic mass. (C) Sagittal reconstructed image showing the large mass with a hypoattenuating non‐enhancing region compatible with necrosis. GB, gallbladder; LLT, liver lobe torsion; ST, stomach; S, spleen.

### 2.2 Surgical Treatment

The dog was premedicated intravenously with midazolam (0.25 mg/kg; Sandoz K.K., Tokyo, Japan) and fentanyl (2 µg/kg; Daiichi Sankyo, Tokyo, Japan). Anaesthesia was induced with propofol (2 mg/kg to effect; Pfizer Inc., NY, USA) and maintained with isoflurane (1.5%–2.0% in oxygen at 2 L/min; DS Pharma, Osaka, Japan) and fentanyl constant rate infusion (2–15 µg/kg/h). Amoxicillin (20 mg/kg IV; Fujita Pharma, Tokyo, Japan) was administered 30 min before surgery and every 90 min intraoperatively, then every 8 h for 48 h postoperatively. Dopamine hydrochloride (5–7 µg/kg/min; Nichi‐Iko Pharmaceutical Co. Ltd., Toyama, Japan), lactated Ringer's solution (3–5 mL/kg/h; Terumo Corporation, Tokyo, Japan) and nafamostat mesilate (0.2 mg/kg/h; Sawai Pharmaceutical Co., Ltd., Osaka, Japan) were administered perioperatively as required.

A midline celiotomy extending from the xiphoid process to the pubis was performed. Abdominal effusion was collected for cytology and bacterial culture. A torsed left medial liver lobe was identified. The falciform ligament and omentum adherent to the mass were carefully separated, and adhesions to the small intestine and spleen were dissected (Figure 2A). The affected lobe was dark and congested with partially pale‐yellow areas, consistent with venous obstruction and ischemic necrosis (Figure 2B). Clockwise torsion was identified at the base of the mass (Figure 2C).

**FIGURE 2:**
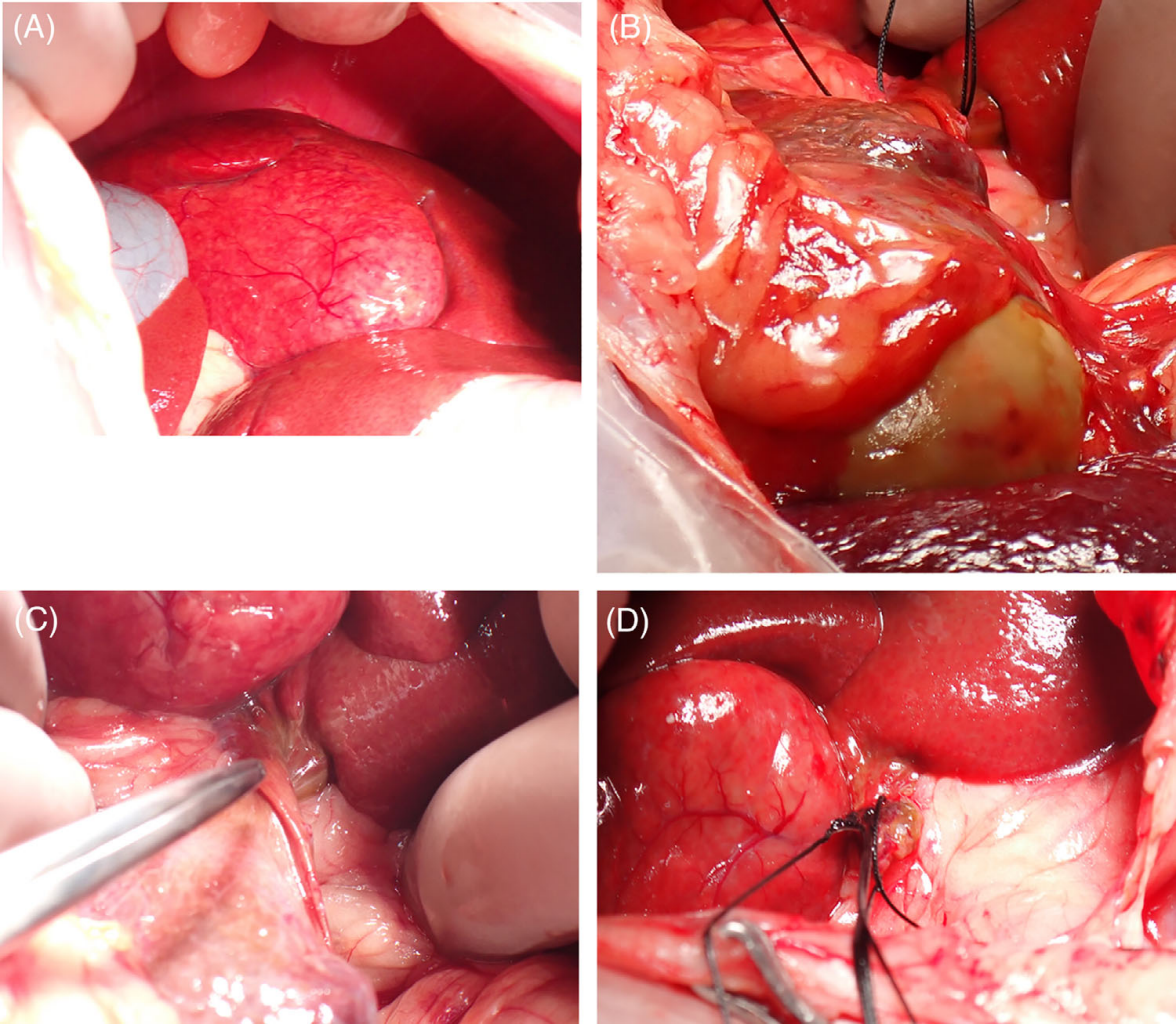
Intraoperative images. (A) Exposure of the torsed left medial liver lobe mass after separation of adherent falciform ligament and omentum. (B) The affected lobe was dark and congested with partially pale‐yellow areas, consistent with venous obstruction and ischemic necrosis. (C) The torsion was identified as clockwise rotation at the base of the mass. (D) After lobectomy, the remaining hepatic parenchyma near the hilus of the left medial liver lobe showed no gross evidence of neoplasia.

The lobe was resected without detorsion to reduce the risk of thromboembolism and systemic release of toxic metabolites. The pedicle was double ligated proximal to the torsed site with 2‐0 monofilament polyglyconate suture and transected. The remaining hepatic parenchyma near the hilus of the left medial lobe had no gross evidence of neoplasia (Figure 2D). No additional gross abnormalities were identified during inspection of the remaining abdominal viscera. An active suction drain was placed, and routine abdominal closure was performed. Resected tissue was submitted for histopathology.

### 2.3 Cytology, Histopathology and Outcome

Cytological analysis of the intraoperatively collected abdominal effusion was consistent with an exudate, characterized by TP in > 30 g/L and total nucleated cell count > 7 × 10^9^ cells/L, with non‐degenerate neutrophils and macrophages. Bacterial culture was negative. The effusion was interpreted as a culture‐negative inflammatory exudate, potentially related to hepatic necrosis and/or tumour‐associated inflammation.

Resected tissue was fixed in 10% neutral buffered formalin for 48 h, embedded in paraffin, sectioned at 4 µm and stained with haematoxylin and eosin. Immunohistochemistry was not performed. Histopathological examination confirmed HCC with extensive necrosis. Neoplastic hepatocytes were arranged in irregular cord‐like to solid patterns, and normal portal triad structures were absent within the lesion. The examined surgical margin was free of neoplastic cells. The adherent falciform ligament contained localised granulation tissue without evidence of malignancy.

The dog recovered uneventfully from anaesthesia. Postoperative analgesia consisted of fentanyl constant rate infusion (1 µg/kg/h) until the following day, followed by buprenorphine (0.01 mg/kg IV q8h) for 72 h. Drain output remained below 1 mL/kg/day, and the drain was removed on postoperative Day 3. Voluntary food intake resumed the day after surgery, and the dog was discharged on postoperative Day 4. No major intraoperative or immediate postoperative complications, including haemorrhage, thrombosis, pancreatitis or sepsis, were identified.

At the 2‐week follow‐up, the dog was doing well, and the preoperative lethargy, inappetence, abdominal pain and dull mentation had resolved. Haematology and serum chemistry showed that ALT and AST had decreased to 166 and 70 U/L, respectively, but remained above the reference intervals. ALP was mildly increased at 215 U/L. CRP and Plt had decreased to 1.6 mg/dL and 508 × 10^3^/µL, respectively, but remained above the reference intervals. TP and Glob had decreased to within the reference intervals.

At 1‐year follow‐up, the dog's body weight had increased by 10%, and the owner reported marked improvement in the dog's activity level. Physical examination was unremarkable. Haematology and serum chemistry showed that ALT, AST, ALP and CRP, which had been increased at the 2‐week postoperative recheck, were within the reference intervals, but only Plt was slightly increased, compared with the reference interval (Table 1). Thoracic radiography and abdominal ultrasonography revealed no evidence of recurrence or metastasis. The dog maintained an excellent quality of life with no clinical abnormalities or complications related to the hepatectomy.

## 3 Discussion

This case illustrates an uncommon presentation of LLT in a small‐sized geriatric dog with concurrent HCC. Although LLT has been reported most frequently in medium‐ to large‐breed dogs, isolated cases in small‐breed dogs have been described (Sonnenfield et al. 2001; Lee et al. 2009). The present case extends the clinical spectrum by documenting the left medial LLT in a Pembroke Welsh Corgi, with CT findings, surgical management, histopathology and 1‐year clinical follow‐up.

Classically, LLT involves rotation of an entire hepatic lobe around its vascular pedicle, usually at or near the hilus. Neoplasia‐associated LLT is rare. Swann and Brown (2001) reported a cat with right medial LLT and concurrent hemoabdomen caused by rupture of an HCC. In dogs, a retrospective series of massive HCC included one dog with torsion of a neoplastic left lateral liver lobe (Liptak et al. 2004), and LLT associated with hepatic hemangiosarcoma has also been reported (Park et al. 2016). In the present dog, however, the axis of rotation was located at the base of the HCC, distal to the left medial lobar hilus, while the proximal hepatic parenchyma near the hilus showed no gross evidence of neoplasia. To our knowledge, this tumour‐base configuration has not previously been described in veterinary LLT and may represent a distinct form of tumour‐associated torsion.

Comparable, although not identical, torsion of pedunculated hepatic tumours has been reported in humans. A pedunculated HCC and pedunculated hepatic hemangiomas have twisted around their vascular stalks and presented as acute abdominal emergencies requiring surgical resection (Reese and Blocker 1994; Darzi et al. 2016; Castañeda Puicón et al. 2020). These reports support a plausible mechanical contribution of a large exophytic or pedunculated mass through altered weight distribution and increased rotational mobility. However, they do not establish a common mechanism, and a causal relationship between HCC and LLT cannot be determined from this single case.

CT was critical for preoperative diagnosis in this atypical case. Radiography and ultrasonography identified a cranial abdominal mass and peritoneal effusion but did not definitively establish LLT. In contrast, CT demonstrated the affected lobe, the non‐enhancing necrotic region and the whirl sign at the base of the mass. In a comparative report of ultrasonographic and CT findings in dogs with LLT, ultrasonography was suggestive of LLT in only one of five cases, whereas CT was fundamental for presurgical diagnosis in all cases (Cordella and Bertolini 2021). The present case further supports the use of CT when LLT is suspected, particularly when the torsion site is peripheral, or the presentation is confounded by a large hepatic mass.

Surgical lobectomy without detorsion was selected to minimize the risk of thromboembolism and systemic release of inflammatory or toxic metabolites from the ischemic lobe. This approach is consistent with previous recommendations for LLT (Swann and Brown 2001; Schwartz et al. 2006). The dog recovered without major complications and had no recurrence of clinical abnormalities at 1 year. This outcome is consistent with reports that dogs with LLT can have an excellent long‐term prognosis if they survive the perioperative period (Schwartz et al. 2006; Scheck 2007). In addition, surgical treatment of massive HCC in dogs has been associated with good tumour control and prolonged survival in appropriate candidates (Liptak et al. 2004).

This report has several limitations. It describes a single dog; therefore, the role of HCC as a predisposing factor for LLT remains speculative. The hepatic tissue proximal to the torsion was not examined histologically, and immunohistochemistry was not performed. Follow‐up extended to only 1 year and included physical examination, blood test, thoracic radiography and abdominal ultrasonography; CT imaging was not performed, and therefore subclinical recurrence or distant metastasis cannot be completely excluded. Larger case series would be needed to determine whether primary hepatic neoplasia is a true risk factor for LLT.

In conclusion, left medial LLT associated with HCC can occur in small‐sized geriatric dogs. CT, particularly identification of the whirl sign, can provide a definitive preoperative diagnosis and guide surgical planning. Concurrent hepatic neoplasia and advanced age should not automatically preclude surgical management when complete resection appears feasible and perioperative stabilisation can be achieved.

## Author Contributions

**Hitomi Shinoda**: conceptualisation (lead), data curation (lead), investigation (lead), methodology (lead), project administration (supporting), visualisation (lead), writing – original draft (lead), writing – review and editing (equal). **Kenji Mori**: data curation (supporting), investigation (supporting), methodology (supporting), visualisation (supporting), writing – review and editing (equal). **Hiroshi Mori**: data curation (supporting), investigation (supporting), methodology (supporting), visualisation (supporting), writing – review and editing (equal). **Kumiko Ishigaki**: project administration (supporting), supervision (supporting), visualisation (supporting), writing – review and editing (equal). **Kazushi Asano**: conceptualisation (supporting), project administration (lead), supervision (lead), writing – original draft (supporting), writing – review and editing (equal).

## Funding

The authors have nothing to report.

## Ethics Statement

Written informed consent was obtained from the dog's owner for publication of anonymised patient information and accompanying images. Data presented in this case report were obtained during routine clinical procedures performed by licensed veterinarians. No experimental procedures were performed, and ethical review was not required.

## Conflicts of Interest

The authors declare no conflicts of interest.

## Data Availability

The data that support the findings of this study are available from the corresponding author upon reasonable request.
